# A new limited memory method for unconstrained nonlinear least squares

**DOI:** 10.1007/s00500-021-06415-8

**Published:** 2021-12-13

**Authors:** Morteza Kimiaei, Arnold Neumaier

**Affiliations:** Fakultät für Mathematik, Universität Wien, Oskar-Morgenstern-Platz 1, A-1090 Wien, Austria

**Keywords:** Black box least squares, Limited memory method, Trust region method, Non-monotone technique

## Abstract

This paper suggests a new limited memory trust region algorithm for large unconstrained black box least squares problems, called **LMLS**. Main features of **LMLS** are a new non-monotone technique, a new adaptive radius strategy, a new Broyden-like algorithm based on the previous good points, and a heuristic estimation for the Jacobian matrix in a subspace with random basis indices. Our numerical results show that **LMLS** is robust and efficient, especially in comparison with solvers using traditional limited memory and standard quasi-Newton approximations.

## Introduction

In this paper, we consider the unconstrained nonlinear least squares problem

1 $$\begin{aligned} \begin{array}{ll} \min &{} f(x){:}{=}\frac{1}{2}\Vert E(x)\Vert ^2_2\\ \mathop {\mathrm{s.t.~}}&{} x\in {\mathbb {R}}^n, \end{array} \end{aligned}$$

with high-dimensional $x\in {\mathbb {R}}^n$ and continuously differentiable $E:{\mathbb {R}}^n\rightarrow {\mathbb {R}}^r$ ($r\ge n$), possibly expensive. However, we assume that no derivative information is available.

### Related work

In recent years, there has been a huge amount of literature on least squares and its applications. Here we just list a useful book and paper:

- Ortega and Rheinboldt (2000) introduced an excellent book, both covering algorithms and their analysis.
- An excellent paper, both covering Levenberg–Marquardt algorithms, quasi-Newton algorithms, and trust region algorithms and their local analysis without non- singularity assumption, has been introduced by Yuan (2011).

Derivative free unconstrained nonlinear black box least squares solvers can be classified in two ways according to how the Jacobian matrix is estimated, and according to whether they are based on line search or on trust region:

- Quasi-Newton approximation. Sorber et al. (2012) introduced **MINLBFGS** (a limited memory BFGS algorithm) and **MINLBFGSDL** (a trust region algorithm using a dogleg algorithm and limited memory BFGS approximation).
- Finite difference approximation. There are many trust region methods using the finite difference method for the Jacobian matrix estimation such as **CoDoSol** and **STRSCNE** by Bellavia et al. (2004, 2012), **NMPNTR** by Kimiaei (2016), **NATRN** and **NATRLS** by Amini et al. (2016, 2016), **LSQNONLIN** from the MATLAB Toolbox, **NLSQERR** (an adaptive trust region strategy) by Deuflhard (2011), and **DOGLEG** by Nielsen (2012). They are suitable for small- and medium-scale problems. Line search methods using the finite difference approximation are **NLEQ** (a damped affine invariant Newton method) by Nowak and Weimann (1990) and **MINFNCG** (a family of nonlinear conjugate gradient methods) by Sorber et al. (2012).

**FMINUNC** by MATLAB Optimization Toolbox is an efficient solver for small- and medium-scale problems. It uses the finite difference method to estimate the gradient vector and the standard quasi-Newton method to estimate the Hessian matrix. In fact, **FMINUNC** disregards the least squares structure and only has access to function values. Nevertheless, it will be shown that **FMINUNC** is more efficient than **LSQNONLIN** using the least squares structure.

To solve the least squares problem (1), trust region methods use linear approximations of the residual vectors to make surrogate quadratic models whose accuracy are increased by restricting their feasible points. These methods use a computational measure to identify whether an agreement between an actual reduction of the objective function and a predicated reduction of surrogate quadratic model function is good or not. If this agreement is good, the iteration is said successful and the trust region radius is expanded; otherwise, the iteration is said unsuccessful and the trust region radius is reduced, for more details see (Conn et al. 2000; Nocedal and Wright 1999).

The efficiency of trust region methods depends on how the trust region radius is updated (see, e.g., Ahookhosh et al. 2013; Amini et al. 2016, 2016); Esmaeili and Kimiaei (2014b, 2014a, 2015); Fan (2006); Fan and Pan (2009, 2010); Kimiaei (2017); Yu and Pu (2008)) and whether non-monotone techniques are applied (see, e.g., Ahookhosh and Amini 2011; Ahookhosh et al. 2015, 2013; Amini et al. 2016, 2016); Deng et al. (1993); Grippo et al. (1986); Grippo and Sciandrone (2007); Kimiaei (2016, 2017); Yu and Pu (2008)). Rounding errors may lead two problems:

(i) The model function may not decrease numerically for some iterations. In this case, if there is no decrease in the function value for such iterations, trust region radii are expanded possibly several times which is an unnecessary expansion for them,
(ii) The model function may decrease numerically but the objective function may not decrease in the cases where iterations are near a valley, deep with a small creek at the bottom and steep sides. In this case, trust region radii are reduced possibly many times, leading to the production of quite a small radius, or even a failure.

Non-monotone techniques can be used in the hope of overcoming the second problem.

### Overview of the new method

We suggest in Sect. 2 a new trust region-based limited memory algorithm for unconstrained black box least squares problems, called **LMLS**. This algorithm uses

- a non-monotone ratio and an adaptive radius formula to quickly reach the minimizer when the valley is narrow;
- a Broyden-like algorithm to get a decrease in the function value when the trust region radius is so small and iteration is unsuccessful;
- a finite difference approximation in a subspace with random basis indices to estimate the Jacobian matrix;
- either a Gauss–Newton or a dogleg algorithm in a subspace with random basis indices to solve the trust region subproblems.

Numerical results for small- to large-scale problems are given in Sect. 3 showing the fact that the new method is suitable for large-scale problems and is more robust and efficient than solvers using limited memory and standard quasi-Newton approximations.

## The trust region method

In this section, we construct an improved trust region algorithm for handling problems in high dimensions:

- In Sect. 2.1 a Gauss-Newton direction in a subspace with random basis indices is introduced.
- In Sect. 2.2 a non-monotone term and an adaptive technique are constructed to quickly reach the minimizer in the presence of a narrow valley.
- In Sect. 2.3 a dogleg algorithm in a subspace with random basis indices is discussed.
- In Sect. 2.4 a Broyden-like technique is suggested based on the old best points.
- In Sect. 2.5 our algorithm using new enhancements is introduced.

We write *J*(*x*) for the Jacobian matrix of the residual vector *E* at *x*. Then the gradient vector is $g(x){:}{=}\nabla f(x){:}{=}J(x)^TE(x)$ and the Hessian matrix is

$$\begin{aligned} G(x){:}{=}J(x)^TJ(x)+\nabla ^2 E(x)^TE(x) \end{aligned}$$

If the residual vector *E*(*x*) is small, the second term in *G*(*x*) is small. Hence, we approximate *G*(*x*) by the Gauss-Newton Hessian matrix $J(x)^TJ(x)$. We define the quadratic surrogate objective function

2 $$\begin{aligned} {\mathcal {Q}}(p){:}{=}\displaystyle \frac{1}{2}\Vert E+Jp\Vert ^2{:}{=}f+p^T g+\displaystyle \frac{1}{2}(Jp)^TJp, \end{aligned}$$

where $f{:}{=}f(x)$, $E{:}{=}E(x)$, $J{:}{=}J(x)$, $g{:}{=}g(x){:}{=}J^TE$. We denote by $A_{:k}$ the *k*th column of a matrix *A*.

A trust region method finds a minimizer of the constrained problem

3 $$\begin{aligned} \begin{array}{ll} \text {min}~{\mathcal {Q}}(p)\\ \text {s.t}\ \ p \in {\mathbb {R}}^{n}\ \ \text{ and }\ \ \Vert p\Vert \le \varDelta ,\\ \end{array} \end{aligned}$$

whose constraint restricts feasible points by the **trust region radius** $\varDelta >0$. This problem is called the **trust region subproblem**. Given a solution *p* of (3), we define the **actual reduction** in the objective function by

4 $$\begin{aligned} df {:}{=} f-f(x+p) \end{aligned}$$

and the **predicted reduction** in the model function by

5 $$\begin{aligned} dq {:}{=} {\mathcal {Q}}(0)-{\mathcal {Q}}(p). \end{aligned}$$

What constitutes an agreement between the actual and predicted reduction around the current iterate *x* must be measured by the **monotone trust region ratio**

6 $$\begin{aligned} \rho {:}{=}\frac{df}{dq}. \end{aligned}$$

If such an agreement is good according to a heuristic formula discussed in Sect. 2.2, the iteration is said **successful**, $x+p$ is accepted as a new point, said a **best point**, and the radius is expanded; otherwise, the iteration is said **unsuccessful** and so the radius is reduced.

### A new subspace Gauss–Newton method

In this subsection, we have two goals: estimating the Jacobian matrix and constructing a Gauss-Newton direction in a subspace with random basis indices.

Let $m_{\mathop {\mathrm{sn}}}$ be the subspace dimension. The Jacobian matrix in a subspace with random basis indices is estimated by a new **subspace random finite difference** called **SRFD** using the following steps:

1. At first, an initial subspace basis indices set is a random subset of $\{1,\ldots ,n\}$ consisting of $m_{\mathop {\mathrm{sn}}}$ members and its complementary is $S^c{:}{=}\{1,\ldots ,n\}\setminus S$.
2. Next, if the complementary of old subspace basis indices set $S^c_{\mathop {\mathrm{old}}}$ is not empty, a new index set ${\mathcal {I}}$ needs to be identified before a new subspace basis indices set is determined. In this case, if ${\mathcal {I}}$ consists of at least $m_{\mathop {\mathrm{sn}}}$ members, ${\mathcal {I}}$ is a random subset of $\{1,\ldots ,m_{\mathop {\mathrm{sn}}}\}$ with the $|S^c_{\mathop {\mathrm{old}}}|$ members; otherwise, it is a permutation of $\{1,\ldots ,|S^c_{\mathop {\mathrm{old}}}|\}$. Then, a new subspace basis indices set is determined by $S{:}{=}S^c_{\mathop {\mathrm{old}}}({\mathcal {I}})$ and its complementary is found by $S^c{:}{=}S^c_{\mathop {\mathrm{old}}}\setminus S$. But if $S^c_{\mathop {\mathrm{old}}}$ is empty, a new subspace basis indices set and its complementary are restarted and chosen in the same way as the initial subspace basis indices set and its complementary, respectively.
3. For any $$i\in S$$,
  the step size is computed by
    $$\begin{aligned} h_i{:}{=} \left\{ \begin{array}{ll} \gamma _s\ \ &{}\hbox {if }x_i=0, \\ \gamma _s(\mathop {\mathrm{sign}}x_i)\max \Big \{|x_i|,\displaystyle \frac{\Vert x\Vert _1}{n}\Big \}\ \ &{} \hbox {otherwise,}\\ \end{array} \right. \end{aligned}$$
    where $0<\gamma _s<1$ is a tiny factor and $\mathop {\mathrm{sign}}x_i$ identifies the sign of $x_i$, taking one of values $-1$ (if $x_i<0$), 0 (if $x_i=0$), and 1 (if $x_i>0$).
  the random approximation coordinate direction *p* discussed in Kimiaei (2020) is used with the difference that its *i*th component is updated by $p_i=p_i+h_i$.
  the new trial residual $E(x+p)$ and the new column $(E(x+p)-E)/h_i$ of the Jacobian matrix are computed.

It is well known that standard quasi-Newton methods are more robust than limited memory quasi-Newton ones, but they cannot handle problems in high dimensions; for standard quasi-Newton methods, see (Dennis and Moré 1977; Dennis and Walker 1981; Nocedal 1992; Schnabel 1989), and for limited memory quasi-Newton methods, see (Liu and Nocedal 1989; Nazareth 1979; Nocedal 1992). On the other hand, finite difference methods are more efficient than standard quasi-Newton ones. Hence, if used in a subspace with random basis indices, they can be more efficient than limited memory quasi-Newton methods for small- up to large-scale problems.

Using *S* and $S^c$ generated and updated by **SRFD**, we construct a new **subspace Gauss-Newton direction** by

7 $$\begin{aligned} (p_{\mathop {\mathrm{sn}}})_{i}{:}{=}\,0 \ \ \text{ for } i\in S^c \text{ and } \ \ (p_{\mathop {\mathrm{sn}}})_S{:}{=}-(J_{:S}^TJ_{:S})^{-1}J_{:S}^TE. \end{aligned}$$

### New non-monotone and adaptive strategies

In this subsection, a new non-monotone term – stronger than the objective function *f* – is constructed and a new adaptive radius formula to update $\varDelta$ is derived from it. They help **LMLS** in finite precision arithmetic to quickly reach the minimizer in the cases where the valley is deep with a small creek at the bottom and steep sides.

Our non-monotone term is updated not only for successful but also for **unsuccessful** iterations that may have happened before a successful iteration is found. This choice is based on an estimated increase in *f* defined below which is updated according to whether a decrease in *f* is found or not. It helps us to generate a somewhat strong non-monotone term when a decrease in *f* is not found and a somewhat weak non-monotone term otherwise. Somewhat strong non-monotone terms increase the chance of finding a point with better function value or at least a point with a little progress in the function value instead of solving trust region subproblems with high computational costs.

We denote by *X* a list of best points and by *F* a list of corresponding function values. Let $m_{\mathop {\mathrm{rs}}}$ be the maximum number of good points saved in *X*. In order to update *X* and *F*, we use **updateXF**. Here we describe how to work it. If $m_{\mathop {\mathrm{rs}}}$ is not exceeded, points with good function values are saved in *X* and their function values in *F*. Otherwise, the worst point and its function value are found and replaced by the best point and its function value, respectively.

Let $\gamma _t\in (0,1)$, $\underline{\gamma }\in (0,1)$, $\gamma ^{{\mathrm{init}}}>0$, and $\overline{\gamma }>1$ be the tuning parameters and let

8 $$\begin{aligned} f_{\max }^k {:}{=} \max _{i=1:m_{\mathop {\mathrm{rs}}}}\{F^k_i\} \ \ \hbox { for all }k=0,1,2,\cdots . \ \ \end{aligned}$$

Before a new non-monotone term is constructed, an estimated increase in *f* needs to be estimated by

9 $$\begin{aligned} \delta _f^k{:}{=} \left\{ \begin{array}{ll} \gamma ^{{\mathrm{init}}}|f^0| &{}\hbox { if }k=0, f^0 \in (0,\infty ), \\ 1 &{}\hbox { if }k=0, f^0\in \{-\infty ,0,\infty \},\\ \displaystyle \frac{1}{\overline{\gamma }}(f^{k-1}-f(x^{k-1}+ p^{k-1})) &{}\hbox { if }k\ge 1, f(x^{k-1}+ p^{k-1}) <f^{k-1},\\ \max (\overline{\gamma }\delta _f^{k-1},\underline{\gamma }(|f(x^{k-1}+ p^{k-1})|+|f_{\max }^k|)) &{}\hbox { if }k\ge 1, f(x^{k-1}+ p^{k-1})\ge f^{k-1}. \end{array}\right. \end{aligned}$$

Accordingly, the new non-monotone formula is defined by

10 $$\begin{aligned} f_{\mathop {\mathrm{nm}}}^k{:}{=}\left\{ \begin{array}{ll} f^0 &{}\hbox { if }k=0, \\ f^k+\delta _f^k &{}\hbox { if }k\ge 1 \end{array}\right. \end{aligned}$$

and the new adaptive radius is constructed by

11 $$\begin{aligned} \varDelta _{\mathop {\mathrm{nm}}}^k {:}{=} \lambda ^k \sqrt{ f_{\mathop {\mathrm{nm}}}^k}, \end{aligned}$$

where $\varDelta _{\mathop {\mathrm{nm}}}^0>0$ is a tuning parameter and $\lambda ^k$ is updated according to

12 $$\begin{aligned} \lambda ^k{:}{=} \left\{ \begin{array}{ll} \sigma _1\lambda ^{k-1},\ \ &{} \hbox {if }{\rho }_{\mathop {\mathrm{nm}}}^{k-1} < \gamma _t, \\ \min (\overline{\lambda },\max (\sigma _2\lambda ^{k-1},\underline{\lambda })), \ \ &{} \hbox {otherwise}.\\ \end{array} \right. \end{aligned}$$

Here $\lambda ^0>0$, $0< \sigma _1< 1 < \sigma _2$, and $\overline{\lambda }> \underline{\lambda }> 0$ are the tuning parameters and the new non-monotone trust region ratio is defined by

13 $$\begin{aligned} \rho _{\mathop {\mathrm{nm}}}^{k-1}{:}{=}\frac{f_{\mathop {\mathrm{nm}}}^{k-1}-f(x^{k-1}+ p^{k-1})}{\widetilde{\mathcal {Q}}^{k-1}(0)-\widetilde{\mathcal {Q}}^{k-1}(p^{k-1})}, \end{aligned}$$

where $p^{k-1}$ is a solution of the following trust region subproblem in a subspace with the random basis indices set *S* by **SRFD**

14 $$\begin{aligned} \begin{array}{ll} \text {min}~\widetilde{\mathcal {Q}}^{k-1}(p){:}{=}\displaystyle \frac{1}{2}\Vert E^{k-1}+J_{:S}^{k-1}p\Vert ^2{:}{=}f^{k-1}+p^T g^{k-1}_S\\ \qquad \qquad \qquad \qquad +\displaystyle \frac{1}{2}(J_{:S}^{k-1}p)^TJ_{:S}^{k-1}p\\ \text {s.t}\ \ p \in {\mathbb {R}}^{m_{\mathop {\mathrm{sn}}}}\ \ \text{ and }\ \ \Vert p\Vert \le \varDelta _{\mathop {\mathrm{nm}}}^{k-1}\\ \end{array} \end{aligned}$$

with $$f^{k-1}{:}{=}f(x^{k-1})$$, $$E^{k-1}{:}{=}E(x^{k-1})$$, $$J^{k-1}_{:S}{:}{=}J_{:S}(x^{k-1})$$, and $$g^{k-1}_S{:}{=}(J_{:S}^{k-1})^TE^{k-1}$$.

### A subspace dogleg algorithm

We define the **Cauchy step** by

15 $$\begin{aligned}&p_{\mathop {\mathrm{c}}}{:}{=}-t^*g_S, \nonumber \\&t^*{:}{=}\displaystyle \mathop {\mathrm{argmin}}\{ \widetilde{\mathcal {Q}}(-tg_S)\mid t\ge 0,~\Vert tg_S\Vert \le \varDelta _{\mathop {\mathrm{nm}}}\}. \end{aligned}$$

The goal is to solve the trust region subproblem (14) such that

16 $$\begin{aligned} \Vert p\Vert \le \varDelta _{\mathop {\mathrm{nm}}}\ \ \text{ and } \ \ \widetilde{\mathcal {Q}}(p)\le \widetilde{\mathcal {Q}}(p_{\mathop {\mathrm{c}}}) \end{aligned}$$

hold. After the subspace Gauss-Newton direction is computed by (7), if it is outside a trust region, a **subspace dogleg algorithm**, called **subDogleg**, is used resulting in an estimated step enforcing (16).

The model function $\widetilde{\mathcal {Q}}$ is reduced by (15) if $dq_{\mathop {\mathrm{sn}}} {:}{=} \widetilde{\mathcal {Q}}(0)-\widetilde{\mathcal {Q}}(p_{\mathop {\mathrm{sn}}})>0$. **subDogleg** first identifies whether $dq_{\mathop {\mathrm{sn}}}>0$ or not. Then we have one of the following cases:

Case 1. If $dq_{\mathop {\mathrm{sn}}}>0$, the **scaled steepest descent step**

17 $$\begin{aligned} p_{\mathop {\mathrm{sd}}}{:}{=}-\displaystyle \frac{g^T_Sg_S}{(J_Sg_S)^T(J_Sg_S)}g_S \end{aligned}$$

is computed. If it is outside the trust region, an estimated solution of (3) is either the Cauchy step computed by (15) or the **dogleg step**

18 $$\begin{aligned} p_{\mathop {\mathrm{dg}}}{:}{=}p_{\mathop {\mathrm{sd}}}+t(p_{\mathop {\mathrm{sn}}}-p_{\mathop {\mathrm{sd}}}); \end{aligned}$$

both of (17) and (18) are on the trust region boundary. Here *t* is found by solving the equation $\Vert p_{\mathop {\mathrm{sd}}}+t(p_{\mathop {\mathrm{sn}}}-p_{\mathop {\mathrm{sd}}})\Vert =\varDelta _{\mathop {\mathrm{nm}}}$. If the condition $dp{:}{=}(p_{\mathop {\mathrm{sd}}})^T(p_{\mathop {\mathrm{sn}}}-p_{\mathop {\mathrm{sd}}})\le 0$ holds, a positive root is computed by

19 $$\begin{aligned} t {:}{=} \frac{-dp+\sqrt{dp^2+\Vert p_{\mathop {\mathrm{sn}}}-p_{\mathop {\mathrm{sd}}}\Vert (\varDelta _{\mathop {\mathrm{nm}}}^2-\Vert p_{\mathop {\mathrm{sd}}}\Vert ^2)}}{\Vert p_{\mathop {\mathrm{sn}}}-p_{\mathop {\mathrm{sd}}}\Vert ^2}\in (0,1). \end{aligned}$$

Otherwise, *t* is computed by

20 $$\begin{aligned} t {:}{=} \frac{\varDelta _{\mathop {\mathrm{nm}}}^2-\Vert p_{\mathop {\mathrm{sd}}}\Vert ^2}{dp+\sqrt{dp^2+\Vert p_{\mathop {\mathrm{sn}}}-p_{\mathop {\mathrm{sd}}}\Vert (\varDelta _{\mathop {\mathrm{nm}}}^2-\Vert p_{\mathop {\mathrm{sd}}}\Vert ^2)}}\in (0,1); \end{aligned}$$

e.g., see Nielsen 2012.

Case 2. If $dq_{\mathop {\mathrm{sn}}}\le 0$, the model function $\widetilde{\mathcal {Q}}$ is convex since the matrix $(J_Sg_S)^T(J_Sg_S)$ is symmetric and positive semidefinite. An estimated solution of (3) is either $p_{\mathop {\mathrm{sd}}}$ computed by (17) if it is inside the trust region or the Cauchy step $p{:}{=}\varDelta _{\mathop {\mathrm{nm}}} (p_{\mathop {\mathrm{sd}}}/\Vert p_{\mathop {\mathrm{sd}}}\Vert )$ according to $p_{\mathop {\mathrm{sd}}}$, otherwise.

### Broyden-like technique

Before a successful iteration is found by a trust region algorithm, the trust region subproblems may be solved many times with high computational cost. Instead, our idea is to use a new algorithm based on the previous best points in the hope of finding a point with good function value.

Whenever **LMLS** cannot decrease the function value, a new Broyden-like technique, called **BroydenLike**, is used in the hope of getting a decrease in the function value. Let $x^1,\ldots ,x^{m_{\mathop {\mathrm{rs}}}}$ be the $m_{\mathop {\mathrm{rs}}}$ best point stored in *X*. Then a point in the affine space spanned by such points has the following form

21 $$\begin{aligned} x_z{:}{=}Xz, \ \ z\in {\mathbb {R}}^{m_{\mathop {\mathrm{rs}}}}, \ \ e^Tz=1, \end{aligned}$$

where $e\in {\mathbb {R}}^{m_{\mathop {\mathrm{rs}}}}$ is a vector all of whose components are one. Given $$B{:}{=}\left( \begin{array}{l} X\\ e\end{array}\right) $$, the linear approximation $E(x_z)\approx Bz$ is used to replace (1) by the surrogate problem

22 $$\begin{aligned} \begin{array}{ll} \min &{}\displaystyle \frac{1}{2}\Vert B z\Vert ^2_2 \\ \mathop {\mathrm{s.t.~}}&{} e^Tz=1. \end{array} \end{aligned}$$

This is a quality constrained convex quadratic problem in $m_{\mathop {\mathrm{rs}}}$ variables and hence can be solved in closed form. Then a QR factorization is made in the form $B=QR$, where *Q* is an orthogonal matrix and *R* is a square upper triangular matrix. By setting $Z{:}{=}R^{-1}$, we make the substitution $z{:}{=}Zy$, define $a^T{:}{=}e^TZ$, and obtain the $m_{\mathop {\mathrm{rs}}}$-dimensional minimal norm linear feasibility problem

23 $$\begin{aligned} \begin{array}{ll} \min &{}\displaystyle \frac{1}{2}\Vert y\Vert ^2_2\\ \mathop {\mathrm{s.t.~}}&{} a^Ty=1 \end{array} \end{aligned}$$

whose solution is $y{:}{=}a/\Vert a\Vert _2$. Hence, a new trial point for the next algorithm is

24 $$\begin{aligned} x^{{\mathrm{trial}}}{:}{=}x_z{:}{=}XR^{-1}a/\Vert a\Vert _2. \end{aligned}$$

**BroydenLike** tries to find a point with better function value when no decrease in *f* is found along *p*. It takes *X*, *F*, *x*, *E*, *B*, *f*, $\delta _f$, and $f_{\mathop {\mathrm{nm}}}$ as input and uses the following tuning parameter:

- $\underline{\gamma }\in (0,1)$ (tiny factor for adjusting $\delta _f$),
- $\overline{\gamma }>1$ (parameter for expanding $\delta _f$),
- $\gamma _s$ (tiny parameter for the finite difference step size), $m_{\mathop {\mathrm{rs}}}$ (memory for affine space),
- $0<\gamma _r<1$ (tiny parameter for adjusting the scaled random directions).

It returns a new value of $\delta _f$ and $f_{\mathop {\mathrm{nm}}}$ (and *f*, *X*, *F*, *E*, *S*, and $J_S$ if a decrease in *f* is found) as output.
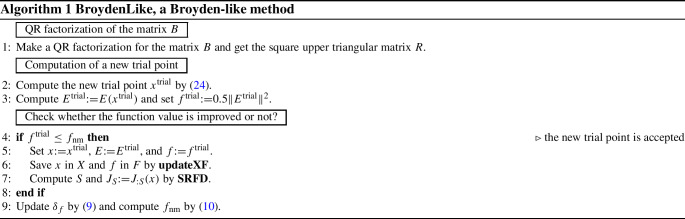


Since the Jacobian matrix may be singular or indefinite, a new point may move toward either a maximum point or saddle point. To remedy this disadvantage, **BroydenLike** does not lead to accept such a point with largest function value.

### A limited memory trust region algorithm

We describe all steps of a new **limited memory algorithm**, called **LMLS** using the new subspace direction (7), the new non-monotone technique (10), the new adaptive radius strategy (11), and **BroydenLike**.

In each iteration, an estimated solution of the trust region subproblem (14) is found. Whenever the condition $\rho _{\mathop {\mathrm{nm}}}\ge \gamma _t$ holds, the iteration is **successful** while updating both the non-monotone term (10) and adaptive radius formula (11), and estimating the Jacobian matrix in a subspace with random basis indices by **SRFD**. Otherwise, the iteration is unsuccessful. In this case, **BroydenLike** is performed in the hope of finding a decrease in the function value. If a decrease in the function value is found, the iteration becomes successful; otherwise, it remains unsuccessful while reducing the radius and updating the non-monotone term (10) until a decrease in the function value is found and the iteration becomes successful.

**LMLS** solves unconstrained nonlinear black box least squares problem. This algorithm takes the initial point $x^0$, and maximal number of function evaluations (nfmax). It uses the following tuning parameters:

- $$m_{\mathop {\mathrm{sn}}}$$ ( subspace dimension),
- $\gamma _t\in (0,1)$ (parameter for trust region),
- $0< \sigma _1< 1 < \sigma _2$ (parameters for updating $\lambda$),
- $\gamma ^{{\mathrm{init}}}$ (parameter for updating the initial $\delta _f$),
- $\underline{\gamma }\in (0,1)$ (tiny factor for adjusting $\delta _f$),
- $\overline{\gamma }>1$ (parameter for expanding $\delta _f$),
- $\underline{\lambda }$ (lower bound for $\lambda$),
- $\overline{\lambda }$ (upper bound for $\lambda$),
- $\gamma _s$ (tiny parameter for adjusting finite difference step sizes),
- $0<\gamma _r<1$ (tiny parameter for adjusting random approximation coordinate directions).

It returns a solution $x^{{\mathrm{best}}}$ of a nonlinear least squares problem as output. 
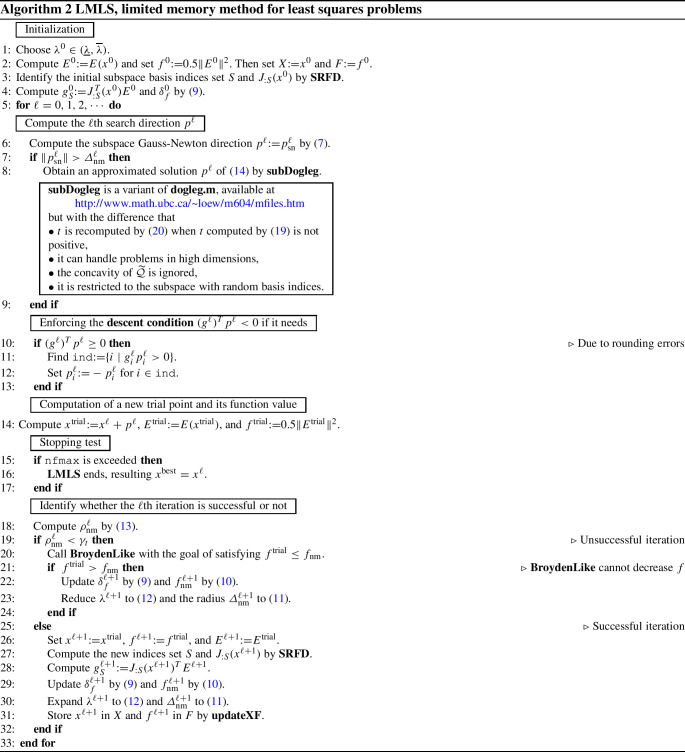


**LMLS** was implemented in MATLAB; the source code is available at

http://www.mat.univie.ac.at/~neum/software/LMLS.

## Numerical results

We updated the test environment constructed by Kimiaei and Neumaier (2019) to use test problems suggested by Lukšan et al. (2018). **LMLS** is compared with unconstrained least squares and unconstrained optimization solvers, for some of which we had to choose options different from the default to make them competitive in the first subsection.

### Codes compared

**Least squares solvers:**

- **CoDoSol** is a solver for constrained nonlinear systems of equations, obtained from

http://codosol.de.unifi.it.

It combines Newton method and a trust region method, see Bellavia et al. (2012). The following option was used

$$\begin{aligned} \mathtt{parms}= & {} [\mathtt{maxit},\mathtt{maxnf},\mathtt{tr},\mathtt{delta},\mathtt{scaling},\\\mathtt{outflag}]= & {} [\mathtt{inf},\mathtt{nfmax},1,-1,0,2]. \end{aligned}$$

Note that $\mathtt{delta}=-1$ means that $\varDelta _0=1$. According to our numerical results, **CoDoSol** was not sensitive for the initial radius; hence, the default was used.

- **STRSCNE** is a solver for constrained nonlinear systems of equations, obtained from

http://codosol.de.unifi.it.

It combines Newton method and a trust region procedure, see Bellavia et al. (2004). The option

$$\begin{aligned} \mathtt{parms}= & {} [\mathtt{maxit},\mathtt{maxnf},\mathtt{delta},\mathtt{outflag}]\\= & {} [\mathtt{inf},\mathtt{nfmax},-1,0] \end{aligned}$$

was used. Note that $\mathtt{delta}=-1$ means that $\varDelta _0=1$. According to our numerical results, **STRSCNE** was not sensitive for the initial radius; hence, the default was used for $\varDelta _0$.

- **NLEQ1** is a damped affine invariant Newton method for nonlinear systems, obtained from
  http://elib.zib.de/pub/elib/codelib/nleq1_m/nleq1.m and suggested by Deuflhard (2011) and written by Nowak and Weimann (1990). The default tuning parameters were used; only $\mathtt{iopt.jacgen}=2$, $\mathtt{iopt.qrank1}=1$, and $\mathtt{wk.fmin = 1e-50}$ were selected.
- **NLEQ2** is the same as **NLEQ1**; only $\mathtt{iopt.qrank1}=0$ was selected.
- **MINLBFGS** is a L-BFGS with line search algorithm, obtained from
  https://www.tensorlab.net/
  and written by Sorber et al. (2012). The default parameters were used, except for *m*. **MINLBFGS1** and **MINLBFGS2** are **MINLBFGS** with $m=\min (n,30)$ and $m=\min (n,100)$, respectively.
- **MINLBFGSDL** is a L-BFGS with dogleg trust region algorithm with the option set $\mathtt{MaxIter = nfmax}$, $\mathtt{TolFun = 1e-50}$, $\mathtt{TolX = 1e-50}$. The default parameters were chosen for $\mathtt{PlaneSearch}$ and *M*. **MINLBFGSDL1**, **MINLBFGSDL2**, **MINLBFG SDL3**, and **MINLBFGSDL4** are **MINLBFGSDL** with
  1. $$\mathtt{PlaneSearch = false}$$ and $$M = \min (30,n)$$,
  2. $$\mathtt{PlaneSearch = false}$$ and $$M = \min (100,n)$$,
  3. $$\mathtt{PlaneSearch = true}$$ and $$M = \min (30,n)$$,
  4. $$\mathtt{PlaneSearch = true}$$ and $$M = \min (100,n)$$.

According to our results, **MINLBFGSDL1** was the best.

- **MINFNCG** is a nonlinear conjugate gradient solver, obtained from
  https://www.tensorlab.net/
  and written by Sorber et al. (2012). We used the following option set
  $$\begin{aligned}&\mathtt{MaxIter = nfmax};\ \ \mathtt{TolFun = 1e-50}; \ \ \\&\mathtt{TolX = 1e-50}. \end{aligned}$$
  The other tuning parameter was $\mathtt{Beta}\in \{\mathtt{HS},\mathtt{HSm},{} \mathtt{PR},\mathtt{FR},\mathtt{PRm},\mathtt{SD}\}$. **MINFNCG1**, **MINFNCG2**, **MINFNCG3**, **MINFNCG4**, **MINFNCG5**, and **MINFNCG6** are **MINFNCG** with $\mathtt{Beta}=\mathtt{HS}$, $\mathtt{Beta}=\mathtt{HSm}$, $\mathtt{Beta}=\mathtt{PR}$, $\mathtt{Beta}=\mathtt{FR}$, $\mathtt{Beta}=\mathtt{PRm}$, and $\mathtt{Beta}=\mathtt{SD}$, respectively.
- **NLSQERR** is a global unconstrained Gauss-Newton method with error oriented convergence criterion and adaptive trust region strategy Deuflhard (2011), obtained from
  http://elib.zib.de/pub/elib/codelib/NewtonLib/index.html
  The following options were used
  $$\begin{aligned}&\mathtt{iniscalx} = 0;\ \ \mathtt{rescalx} = 0; \ \ \mathtt{xthrsh} = \mathop {\mathrm{ones}}(n, 1); \\&\mathtt{xtol} = \mathtt{1.e-50};\ \ \mathtt{ftol} = \mathtt{1.e-50};\ \ \mathtt{kmax} = \mathtt{nfmax};\\&\mathtt{printmon} = 2;\ \ \mathtt{printsol} = 1;\ \ \mathtt{fid} = 1; \ \ \\&\mathtt{numdif} = 1; \\&\mathtt{lambda0} = \mathtt{eps}; \mathtt{lambdamin} = \mathtt{1e-50}; \\&\mathtt{ftol} = \mathtt{1.e-50}. \end{aligned}$$
- **NMPNTR**, non-monotone projected Newton trust region method, is a bound constrained solver Kimiaei (2016). **NMPNTR1**, **NMPNTR2**, **NMPNTR3**, and **NMPNTR4** are **NMPNTR** with $\varDelta _0=1$, $\varDelta _0=10$, $\varDelta _0=100$, and $\varDelta _0=500$, respectively. According to our results, **NMPNTR2** was the best.
- **NATRN** is a non-monotone trust region algorithm Amini et al. (2016) using the full finite difference approximation. The subproblem was solved in the same way as **LMLS**. **NATRN1**, **NATRN2**, **NATRN3**, and **NATRN4** are **NATRN** with $\varDelta _0=1$, $\varDelta _0=10$, $\varDelta _0=100$, and $\varDelta _0=500$, respectively. According to our results, **NATRN1** had the best performance.
- **NATRLS** is a non-monotone line search and trust region algorithm Amini et al. (2016) using the full finite difference approximation. The subproblem was solved in the same way as **LMLS**. **NATRLS1**, **NATRLS2**, **NATRLS3**, and **NATRLS4** are **NATRLS** with $\varDelta _0=1$, $\varDelta _0=10$, $\varDelta _0=100$, and $\varDelta _0=500$, respectively. According to our results, **NATRLS1** had the best performance.
- **LSQNONLIN1**, obtained from the MATLAB Optimization Toolbox at,
  https://de.mathworks.com/help/optim/ug/lsqnonlin.html
  is a nonlinear least squares solver with the following options:
  options = optimoptions(@lsqnonlin,‘Algorithm’, ‘levenberg-marquardt’, ‘Fin- iteDifferenceType’,‘forward’, ‘MaxIter’, Inf,‘MaxFunEvals’, nfmax, ‘TolX’, 0,‘Specify ObjectiveGradient’,‘false’).
- **LSQNONLIN2**, obtained from the MATLAB Optimization Toolbox at,
  https://de.mathworks.com/help/optim/ug/lsqnonlin.html
  is a nonlinear least squares solver with the following options:
  options = optimoptions(@lsqnonlin,‘Algorithm’, ‘trust-region reflective’, ‘FiniteDifferenceType’,‘forward’, ‘MaxIter’, Inf,‘MaxFunEvals’, nfmax, ‘TolX’, 0,‘SpecifyObjectiveGradient’,‘false’).
- **DOGLEG** is Powell’s dogleg method for least squares problems, which is the best algorithm from the toolbox of immoptibox.zip Nielsen (2012), available at
  http://www2.imm.dtu.dk/projects/immoptibox/
  The following option was used
  $$\begin{aligned} \mathtt{opts}= & {} [\varDelta _0,\mathtt{tolg},\mathtt{tolx},\mathtt{tolr},\mathtt{maxeval}]\\= & {} [\varDelta _0,\mathtt{1e-50},\mathtt{1e-50},\mathtt{1e-50},\mathtt{nfmax}]. \end{aligned}$$
  **DOGLEG1**, **DOGLEG2**, **DOGLEG3**, and **DOGLEG4** are **DOGLEG** with $\varDelta _0=1$, $\varDelta _0=10$, $\varDelta _0=100$, and $\varDelta _0=500$, respectively. The best version was **DOGLEG1**.

**Unconstrained solvers:**

- **FMINUNC**, obtained from the MATLAB Optimization Toolbox at
  https://ch.mathworks.com/help/optim/ug/fminunc.html,
  is a standard quasi-Newton algorithm. We used **FMINUNC** with
  opts = optimoptions(@fminunc),‘Algorithm’,‘quasi-newton’, ‘Display’, ‘Iter’, ‘MaxIter’,Inf,‘MaxFunEvals’, nfmax, ‘TolX’, 0,‘TolFun’,0,‘ObjectiveLimit’,-1e-50).
- **FMINUNC1** is **FMINUNC** with the limited memory quasi-Newton approximation by Liu and Nocedal (1989). It were added to **FMINUNC** by the present authors. The option set for it was used the same as **FMINUNC**; only the memory $m=10$ was added to the option set.

### Default for tuning parameters of **LMLS**

The tuning parameters for our new method (**LMLS**) are chosen as

$$\begin{aligned} \boxed {\begin{array}[c]{llll} m_{\mathop {\mathrm{nm}}}=10;&{} m_{\mathop {\mathrm{rs}}}=10;&{} \gamma _r=10^{-30}; &{} \gamma _p=5\varepsilon _m;\\ \gamma _s=\sqrt{\varepsilon _m};&{} \gamma _t=0.1;&{} \sigma _1=0.5;&{} \lambda ^0=1; \\ \sigma _2=1; &{}\gamma ^{{\mathrm{init}}}=10^{-8};&{} \gamma _m=10^{-30};&{} \underline{\lambda }=10^{-4};\\ \varDelta ^0=10;&{} \varDelta ^{\min }=10^{-6};&{} \overline{\lambda }=10^5.&{}\\ \end{array}} \end{aligned}$$

The remaining tuning parameter $m_{\mathop {\mathrm{sn}}}$ is varied in the experiment: **LMLS1**, **LMLS2**, **LMLS3**, and **LMLS4** are **LMLS** with $m_{\mathop {\mathrm{sn}}}=3$, $m_{\mathop {\mathrm{sn}}}=\min (10,n)$, $m_{\mathop {\mathrm{sn}}}=\min (30,n)$, and $m_{\mathop {\mathrm{sn}}}=\min (100,n)$, respectively.

### Test problems used, initial point, and stopping tests

Test problems suggested by Lukšan et al. (2018) are classified in Table 1 according to whether they are **overdetermined** ($r>n$) or not ($r=n$). A shifted point for these problems is done like Kimiaei and Neumaier (2020) as $\chi _i {:}{=} (-1)^{i-1}2/(2+i)$ for all $i=1,\ldots ,n$. This means that the initial point is chosen by $x^0_i=\chi _i$ for all $i=1,\ldots ,n$ and the initial function value is computed by $f^0{:}{=}f(x^0)$, while the other function values are computed by $f^\ell {:}{=}f(x^{\ell }+\chi )$ for all $\ell \ge 1$.

**Table 1:** A classification of test problems

Dimensions *n*	3	5	10	16	30	50	100	300	500	1000	5000	10,000
Total number of least squares problems ($r\ge n$)	49	69	76	83	83	83	83	83	83	83	83	83
Number of square problems ($r=n$)	43	53	55	62	62	62	62	62	62	62	62	62
Number of least squares problems with $r>n$	6	16	21	21	21	21	21	21	21	21	21	21

We denote $\mathtt{nf}$ and $\mathtt{msec}$ as the number of function evaluations and time in milliseconds, respectively. nfmax and secmax are the upper bounds for them, chosen as

$$\begin{aligned} \mathtt{nfmax}\in \left\{ \begin{array}{ll} \{10n,50n,100n,500n\} &{}\hbox { if }1\le n \le 100, \\ \{10n,50n,100n\} &{}\hbox { if }101\le n \le 1000\\ \{10n,100n\} &{}\hbox { if }1001\le n \le 10000 \end{array}\right. \end{aligned}$$

and

$$\begin{aligned} \mathtt{secmax}{:}{=} \left\{ \begin{array}{ll} 300 &{}\hbox { if }1\le n \le 100, \\ 800 &{}\hbox { if }101\le n \le 10000.\\ \end{array}\right. \end{aligned}$$

Denote by $f^0$ the function value of the starting point (common to all solvers), by $f^{so}$ the best point found by the solver *so*, and by $f^{\mathop {\mathrm{opt}}}$ the best point known to us. Then, if the target accuracy satisfies

$$\begin{aligned} q^{so}&{:}{=}&(f^{so}-f^{\mathop {\mathrm{opt}}})/(f^0-f^{\mathop {\mathrm{opt}}})\\\le & {} \left\{ \begin{array}{ll} 10^{-8} &{}\hbox { if }1\le n \le 100, \\ 10^{-3} &{}\hbox { if }101\le n \le 10000, \end{array}\right. \end{aligned}$$

then the problem is solved by the solver *so*. Otherwise, the problem is unsolved by it; either nfmax or secmax is exceeded, or the solver fails.

### The efficiency and robustness of a solver

For a given collection *S* of solvers, the strength of a solver $so \in S$—relative to an ideal solver that matches on each problem the best solver—is measured, for any given cost measure $c_s$ by the number, $e_{so}$ defined by

$$\begin{aligned} e_{so}{:}{=}\left\{ \begin{array}{ll} \displaystyle \frac{\displaystyle \min _{s\in S}c_{s}}{c_{so}},~~~&{} \quad \hbox {if the solver }so\hbox { solves the problem},\\ 0,~~~&{} \quad \hbox {otherwise}, \end{array} \right. \end{aligned}$$

called the **efficiency** of the solver *so* with respect to this cost measure. Two cost measures nf and msec are used.

The **robustness** of a solver is how many test problems it can solve. Efficiency and robustness are two adequate tools to determine which solver is competitive. In fact, the robustness of a solver is more important than its efficiency. We use two different performance plots in terms of the robustness and efficiency of solvers:

- The first performance plot is the data profile by Moré and Wild (2009) for nf/(best nf) and msec/(best msec) as well; but it is the percentage of problems solved within the number of function evaluations and time in milliseconds.
- The second performance plot is the performance profile by Dolan and Moré (2002) for nf/(best nf) and msec/(best msec); the percentage of problems solved within a factor $\tau$ of the best solvers.

All tables and data/performance profiles are given in Sects. 4.2–4.4. In Sects. 3.5-3.7, we summarize them as two new performance plots.

### Small scale: $n\in [1,100]$

**Fig. 1:**
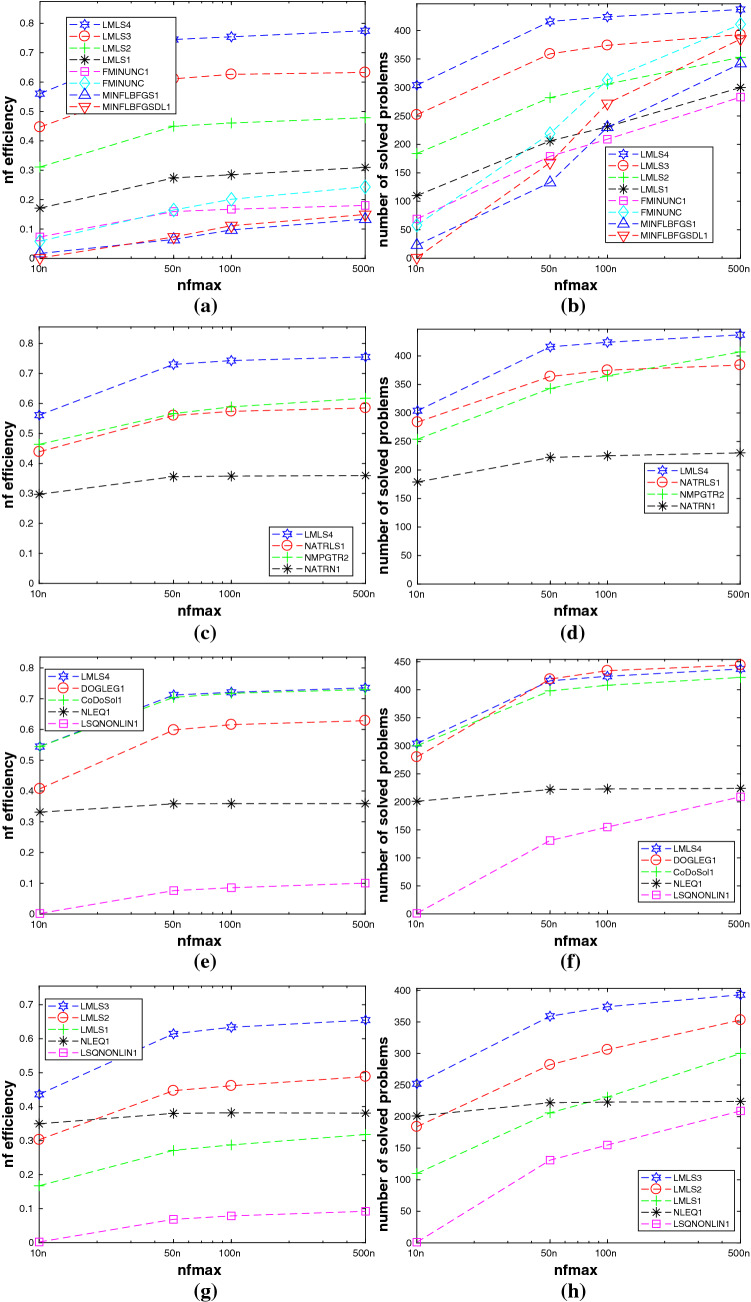
Performance plots for small-scale problems. **a**–**b**: A comparison of limited memory solvers, **c**–**d**: A comparison among **LMLS** in a full subspace with random basis indices and solvers using other non-monotone and adaptive radius techniques, **e**–**f**: A comparison among **LMLS** in a full subspace with random basis indices and other famous solvers, **g**–**h**: A comparison among low-dimensional **LMLS1**, **LMLS2**, **LMLS3**, and **NLEQ1** and **LSQNONLIN1** using full estimated Jacobian

A comparison among **LMLS1**, **LMLS2**, **LMLS3**, **LMLS4**, and solvers using quasi-Newton is shown in Subfigures (a) and (b) of Fig. 1, so that

- **LMLS4** using the full estimated Jacobian matrix is the best in terms of the number of solved problems and the nf efficiency;
- **LMLS3**, **LMLS2**, and **LMLS1** are more efficient than solvers using quasi-Newton approximation (**FMINUNC**, **FMINUNC1**, **MINFLBFGS1**, and **MINFLBFGSDL1**) in terms of the nf efficiency;
- **FMINUNC** and **MINFLBFGSDL1** are comparable with **LMLS3**—only for very large budget—in terms of the number of solved problems but **LMLS3**, **LMLS2**, and **LMLS1** are more efficient than **FMINUNC** and **MINFLBFGSDL1** in terms of the nf efficiency not only for very large budget but also for small up to large budgets.

To determine whether our new non-monotone and adaptive radius strategies are effective or not, we compare **LMLS4** with solvers using other non-monotone and adaptive radius strategies, shown in Subfigures (c) and (d) of Fig. 1. All solvers use the full Jacobian matrix and the trust region subproblems are solved by the same algorithm. As can be seen, **LMLS4** is much more efficient and robust than **NATRLS1**, **NMPGTR2**, and **NATRN1** in terms of the number of solved problems and the nf efficiency.

We compare **LMLS4** with four famous solvers **LSQNONLIN1**, **CoDoSol1**, **NLEQ1**, and **DOGLEG1** shown in Subfigures (e) and (f) of Fig. 1. It is seen that **LMLS4** and **CoDoSol1** are the two best solvers in terms of the nf efficiency while **LMLS4** and **DOGLEG1** are the two best solvers in terms of the number of solved problems.

Another comparison is among **LMLS3**, **LMLS2**, and **LMLS1** using the Jacobian matrix in an adaptive subspace basis indices set and **LSQNONLIN1** and **NLEQ1** using the full Jacobian matrix. We conclude from Subfigures (g) and (h) of Fig. 1 that

- **LMLS3** is the best in terms of the number of solved problems and the nf efficiency;
- **LMLS2** is the second best solver in terms of the number of solved problems and the nf efficiency for medium, large, and very large budgets;
- **LMLS1** with lowest subspace dimension is more efficient than **LSQNONLIN1** in terms of the number of solved problems and the nf efficiency; even it is more efficient than **NLEQ1** for very large budget in terms of the nf efficiency.

As a result, **LMLS** is competitive for small-scale problems in comparison with the state-of-the-art solvers.

### Medium scale: $n\in [101,1000]$

**Fig. 2:**
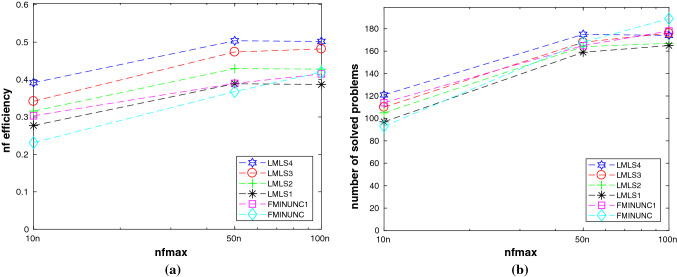
**a**–**b**: Performance plots for medium-scale problems

In this subsection, we compare **LMLS1**, **LMLS2**, **LMLS3**, and **LMLS4** using the estimated Jacobian matrices in a subspace with random basis indices with **FMINUNC** using standard BFGS approximations and **FMINUNC1** using limited memory BFGS ones (Fig. 2).

From Subfigures (a) and (b) of Fig. 1, we conclude that

- **LMLS4**, **LMLS3**, and **LMLS2** are the three best solvers in terms of the nf efficiency, respectively;
- **LMLS4** is the best solver in terms of the number of solved problems; only **FMINUNC** is the best for large budget.

**Fig. 3:**
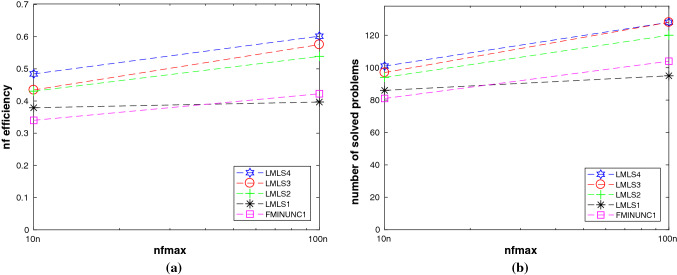
**a**–**b**: Performance plots for large-scale problems

**Table 2:** Results for small-scale and small budget

Stopping test:	$q^{so}\le$ 1e-08, $\mathtt{sec}\le$ 300, $\mathtt{nf}\le$ 10*n									
367 of 526 problems without bounds solved									Mean efficiency in %	
dim$\in$[1,100]						# of anomalies			For cost measure	
Solver		Solved	#100	!100	$T_{\text {mean}}$	#n	#t	#f	nf	msec
**LMLS4**	**lmtr4**	**304**	**204**	15	52	222	0	0	**53**	**46**
**CoDoSol1**	**codo1**	300	197	**30**	56	219	0	7	52	40
**NATRLS1**	**natrs1**	284	63	8	67	242	0	0	40	30
**DOGLEG1**	**dogleg1**	280	70	11	98	246	0	0	38	22
**NMPGTR2**	**nmpg2**	254	172	8	55	272	0	0	43	31
**LMLS3**	**lmtr3**	252	156	4	53	274	0	0	42	35
**NLEQ1**	**nleq1**	201	42	**30**	77	145	0	180	33	16
**LMLS2**	**lmtr2**	184	91	7	75	342	0	0	29	22
**NATRN1**	**natrn1**	179	54	11	89	347	0	0	26	18
**LMLS1**	**lmtr1**	110	47	20	112	416	0	0	16	10
**STRSCNE1**	**strs1**	70	69	0	**28**	107	0	349	13	7
**FMINUNC1**	**func1**	69	2	0	123	455	0	2	6	4
**FMINUNC**	**func**	58	2	0	139	468	0	0	5	4
**MINFLBFGS1**	**lbfgs1**	23	0	0	227	449	0	54	1	0
**LSQNONLIN1**	**lsqn1**	1	1	1	50	525	0	0	0	0
**MINFLBFGSDL1**	**minlbfgs1**	1	0	0	30	525	0	0	0	0
**MINFNCG1**	**MINFNCG1**	0	0	0	−	514	0	12	0	0

**Table 3:** Results for small-scale and medium budget

Stopping test:	$q^{so}\le$ 1e-08, $\mathtt{sec}\le$ 300, $\mathtt{nf}\le$ 50*n									
474 of 526 problems without bounds solved									Mean efficiency in %	
dim$\in$[1,100]						# of anomalies			For cost measure	
Solver		Solved	#100	!100	$T_{\text {mean}}$	#n	#t	#f	nf	msec
**DOGLEG1**	**dogleg1**	**419**	80	17	148	107	0	0	55	30
**LMLS4**	**lmtr4**	416	**228**	17	108	110	0	0	**66**	**59**
**CoDoSol1**	**codo1**	398	226	**52**	90	107	0	21	65	54
**NATRLS1**	**natrs1**	364	79	21	81	162	0	0	51	39
**LMLS3**	**lmtr3**	359	177	8	124	167	0	0	55	46
**NMPGTR2**	**nmpg2**	343	191	22	79	183	0	0	53	40
**LMLS2**	**lmtr2**	282	111	16	197	244	0	0	40	29
**NATRN1**	**natrn1**	222	63	17	117	304	0	0	31	22
**NLEQ1**	**nleq1**	222	42	30	77	122	0	182	35	18
**FMINUNC**	**func**	219	4	3	167	292	0	15	13	11
**LMLS1**	**lmtr1**	206	49	21	253	320	0	0	24	15
**FMINUNC1**	**func1**	179	8	7	177	345	0	2	13	10
**MINFLBFGSDL1**	**minlbfgs1**	168	0	0	286	358	0	0	5	5
**MINFLBFGS1**	**lbfgs1**	133	0	0	200	339	0	54	5	4
**LSQNONLIN1**	**lsqn1**	131	1	1	314	395	0	0	5	4
**STRSCNE1**	**strs1**	72	69	0	**28**	1	0	453	13	8
**MINFNCG1**	**MINFNCG1**	47	0	0	698	458	0	21	1	1

### Large scale: $n\in [1001,10000]$

In this subsection, we compare **LMLS1**, **LMLS2**, **LMLS3**, **LMLS4** using the estimated Jacobian matrices in a subspace with random basis indices with **FMINUNC1** using limited memory BFGS approximations.

In terms of the nf efficiency and the number of solved problems, Subfigures (a) and (b) of Fig. 3 result in the fact that

- **LMLS4**, **LMLS3**, and **LMLS2** are the three best solvers, respectively;
- **LMLS1** with lowest subspace dimension is more efficient than **FMINUNC1** for small budget while **FMINUNC1** is more efficient than **LMLS1** for large budget.

## Additional material for LMLS

### Summarizing tables

In all tables, efficiencies are given as percentages, rounded (towards zero) to integers. Larger efficiencies imply a better average behavior, while a zero efficiency indicates failure.

#100 is the total number of problems for which the solver *so* was best with respect to nf ($e_{so}=1=100\%$). !100 is the total number of problems solved for which the solver *so* was better than all other solvers with respect to nf.

We denote the time in seconds without the setup time for the objective function by sec. In tables, a sign

- *n* indicates that nf $\ge \mathtt{nfmax}$ was reached.
- *t* indicates that sec $\ge \mathtt{secmax}$ was reached.
- *f* indicates that the algorithm failed for other reasons.

$T_{\mathop {\mathrm{mean}}}$ is the mean of the time in seconds needed by a solver to solve the test problems chosen from the list of test problems ${\mathcal {P}}$, ignoring the times for unsolved problems. It can be a good measure when solvers have approximately the same number of solved problems.

### Tables and data/performance profiles for $1\le n\le 100$

**Fig. 4:**
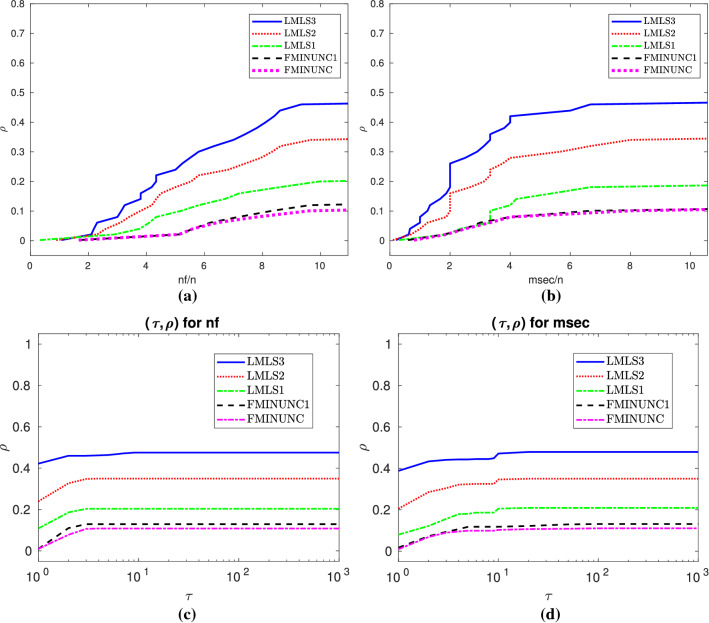
**a** and **b**: Data profiles for nf/(best nf) and msec/(best msec), respectively. $\rho$ designates the fraction of problems solved within the number of function evaluations and time in milliseconds used by the best solver. Problems solved by no solver are ignored. **c**–**d**: Performance profiles for nf/(best nf) and msec/(best msec), respectively. $\rho$ designates the fraction of problems solved within the number of function evaluations and time in milliseconds used by the best solver. Problems solved by no solver are ignored

**Fig. 5:**
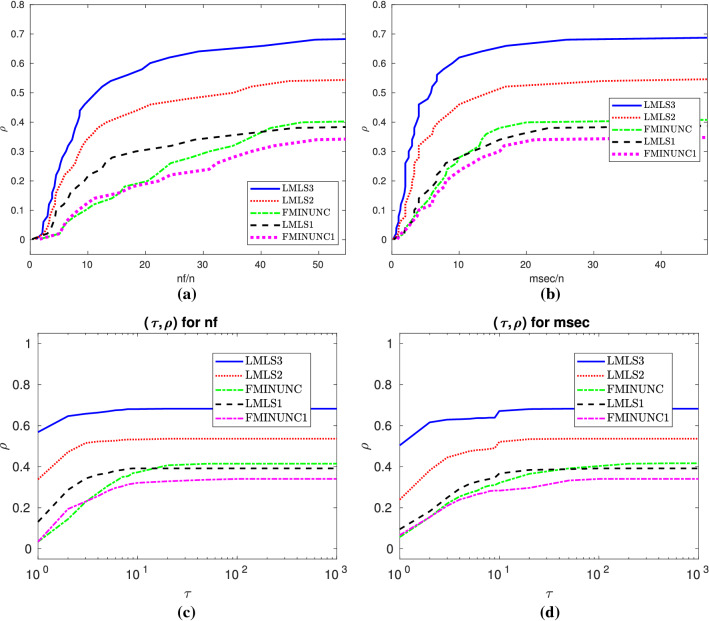
Details as in Fig. 4

**Fig. 6:**
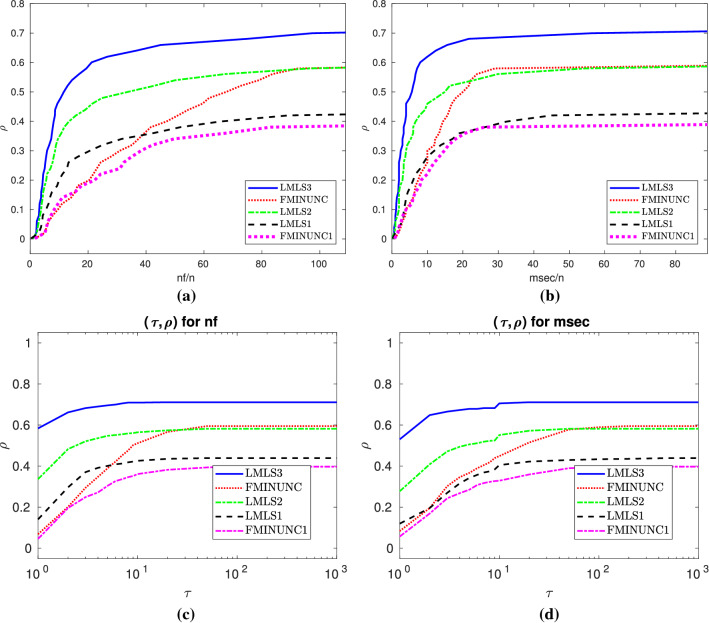
Details as in Fig. 4

**Fig. 7:**
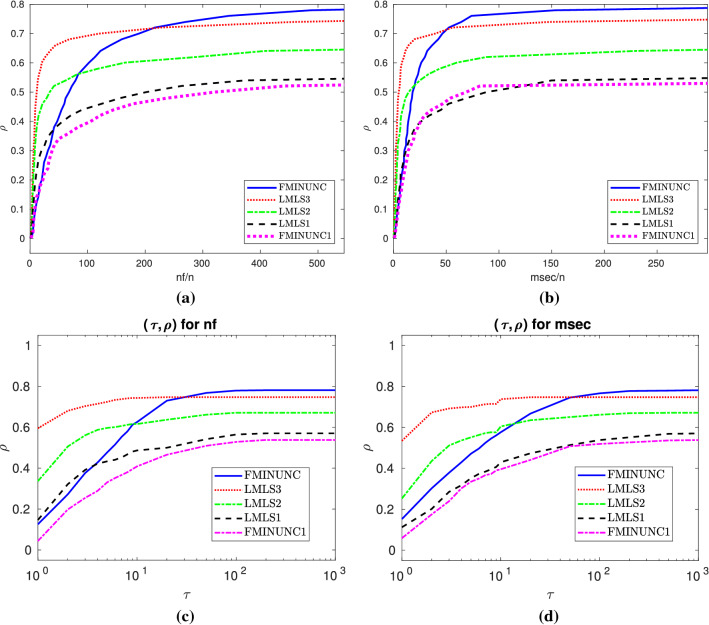
Details as in Fig. 4

This section contains Tables 2, 3, 4, 5 and Figs. 4, 5, 6, 7, summaries of which were discussed in Sect 3.5.

**Table 4:** Results for small-scale and large budget

Stopping test:	$q^{so}\le$ 1e-08, $\mathtt{sec}\le$ 300, $\mathtt{nf}\le$ 100*n									
493 of 526 problems without bounds solved									Mean efficiency in %	
dim$\in$[1,100]						# of anomalies			For cost measure	
Solver		Solved	#100	!100	$T_{\text {mean}}$	#n	#t	#f	nf	msec
**DOGLEG1**	**dogleg1**	**434**	82	19	167	92	0	0	56	31
**LMLS4**	**lmtr4**	424	**229**	17	113	102	0	0	**67**	**59**
**CoDoSol1**	**codo1**	408	227	**53**	103	88	0	30	66	54
**NATRLS1**	**natrs1**	375	87	27	104	151	0	0	53	39
**LMLS3**	**lmtr3**	374	177	9	150	152	0	0	56	48
**NMPGTR2**	**nmpg2**	365	196	25	103	160	0	1	54	40
**FMINUNC**	**func**	313	6	4	351	170	0	43	16	14
**LMLS2**	**lmtr2**	306	106	12	257	220	0	0	41	31
**MINFLBFGSDL1**	**minlbfgs1**	272	2	2	453	244	0	10	9	8
**LMLS1**	**lmtr1**	231	53	24	437	295	0	0	25	17
**MINFLBFGS1**	**lbfgs1**	230	0	0	349	232	0	64	8	6
**NATRN1**	**natrn1**	225	65	19	121	301	0	0	32	22
**NLEQ1**	**nleq1**	223	43	30	79	121	0	182	35	17
**FMINUNC1**	**func1**	209	8	6	327	297	0	20	14	12
**LSQNONLIN1**	**lsqn1**	155	1	1	374	371	0	0	6	4
**MINFNCG1**	**MINFNCG1**	136	0	0	524	365	0	25	2	2
**STRSCNE1**	**strs1**	72	69	0	**28**	1	0	453	13	7

**Table 5:** Results for small-scale and very large budget

Stopping test:	$q^{so}\le$ 1e-08, $\mathtt{sec}\le$ 300, $\mathtt{nf}\le$ 500*n									
509 of 526 problems without bounds solved									Mean efficiency in %	
dim$\in$[1,100]						# of anomalies			For cost measure	
Solver		Solved	#100	!100	$T_{\text {mean}}$	#n	#t	#f	nf	msec
**DOGLEG1**	**dogleg1**	**444**	79	18	220	82	0	0	57	31
**LMLS4**	**lmtr4**	437	**229**	18	159	89	0	0	**68**	**58**
**CoDoSol1**	**codo1**	422	226	**54**	172	61	0	43	67	55
**FMINUNC**	**func**	411	8	7	761	20	0	95	19	17
**NMPGTR2**	**nmpg2**	407	194	25	378	110	1	8	56	42
**LMLS3**	**lmtr3**	393	178	9	343	133	0	0	56	47
**MINFLBFGSDL1**	**minlbfgs1**	385	6	6	723	69	0	72	11	12
**NATRLS1**	**natrs1**	384	89	31	143	142	0	0	54	41
**LMLS2**	**lmtr2**	353	107	14	998	173	0	0	42	31
**MINFLBFGS1**	**lbfgs1**	342	4	4	973	56	0	128	10	9
**LMLS1**	**lmtr1**	300	53	25	1302	226	0	0	27	17
**MINFNCG1**	**MINFNCG1**	283	0	0	990	194	0	49	3	4
**FMINUNC1**	**func1**	283	8	7	534	205	0	38	15	13
**NATRN1**	**natrn1**	230	63	17	178	296	0	0	32	22
**NLEQ1**	**nleq1**	224	42	30	82	120	0	182	35	18
**LSQNONLIN1**	**lsqn1**	209	1	1	690	316	1	0	6	5
**STRSCNE1**	**strs1**	72	69	0	**29**	1	0	453	13	6

**Table 6:** Results for medium-scale and small budget

Stopping test:	$q^{so}\le$ 0.001, $\mathtt{sec}\le$ 800, $\mathtt{nf}\le$ 10*n									
154 of 249 problems without bounds solved									Mean efficiency in %	
dim$\in$[101,1000]						# of anomalies			For cost measure	
Solver		Solved	#100	!100	$T_{\text {mean}}$	#n	#t	#f	nf	msec
**LMLS4**	**lmls4**	**119**	**49**	**48**	13738	129	1	0	**37**	**37**
**FMINUNC1**	**func1**	114	40	14	**11271**	133	2	0	29	35
**LMLS3**	**lmtr3**	110	30	28	15401	138	1	0	34	28
**LMLS2**	**lmtr2**	105	22	21	15006	143	1	0	31	20
**LMLS1**	**lmtr1**	97	15	15	18207	151	1	0	27	21
**FMINUNC**	**func**	93	26	0	13702	154	2	0	22	24

**Table 7:** Results for medium-scale and budget

Stopping test:	$q^{so}\le$ 0.001, $\mathtt{sec}\le$ 800, $\mathtt{nf}\le$ 50*n									
188 of 249 problems without bounds solved									Mean efficiency in %	
dim$\in$[101,1000]						# of anomalies			For cost measure	
Solver		Solved	#100	!100	$T_{\text {mean}}$	#n	#t	#f	nf	msec
**LMLS4**	**lmtr4**	**169**	**58**	**56**	11582	78	2	0	**50**	46
**LMLS3**	**lmtr3**	168	48	47	12980	79	2	0	47	37
**FMINUNC**	**func**	168	32	5	11064	78	3	0	36	42
**FMINUNC1**	**func1**	165	43	16	**10503**	81	3	0	39	**50**
**LMLS2**	**lmtr2**	164	16	15	15614	84	1	0	43	25
**LMLS1**	**lmtr1**	159	20	20	15399	88	2	0	38	28

**Table 8:** Results for medium-scale and large budget

Stopping test:	$q^{so}\le$ 0.001, $\mathtt{sec}\le$ 800, $\mathtt{nf}\le$ 100*n									
198 of 249 problems without bounds solved									Mean efficiency in %	
dim$\in$[101,1000]						# of anomalies			For cost measure	
Solver		Solved	#100	!100	$T_{\text {mean}}$	#n	#t	#f	nf	msec
**FMINUNC**	**func**	**189**	44	18	14603	55	3	2	41	48
**FMINUNC1**	**func1**	178	46	20	**12133**	66	3	2	41	**52**
**LMLS3**	**lmtr3**	176	39	37	16832	71	2	0	48	36
**LMLS4**	**lmtr4**	174	**53**	**51**	18938	73	2	0	**50**	47
**LMLS2**	**lmtr2**	167	24	24	18182	81	1	0	42	24
**LMLS1**	**lmtr1**	165	20	20	28711	84	0	0	38	26

### Tables and data/performance profiles for $101 \le n \le 1000$

**Fig. 8:**
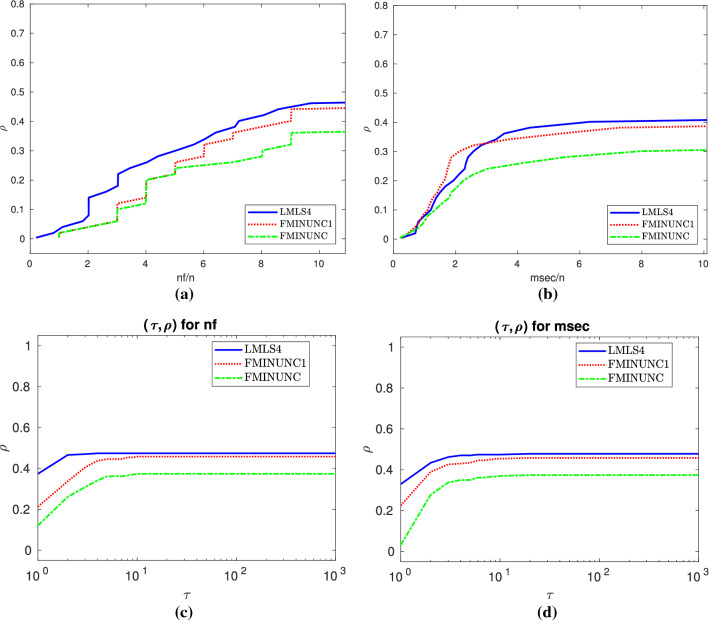
Details as in Fig. 4

**Fig. 9:**
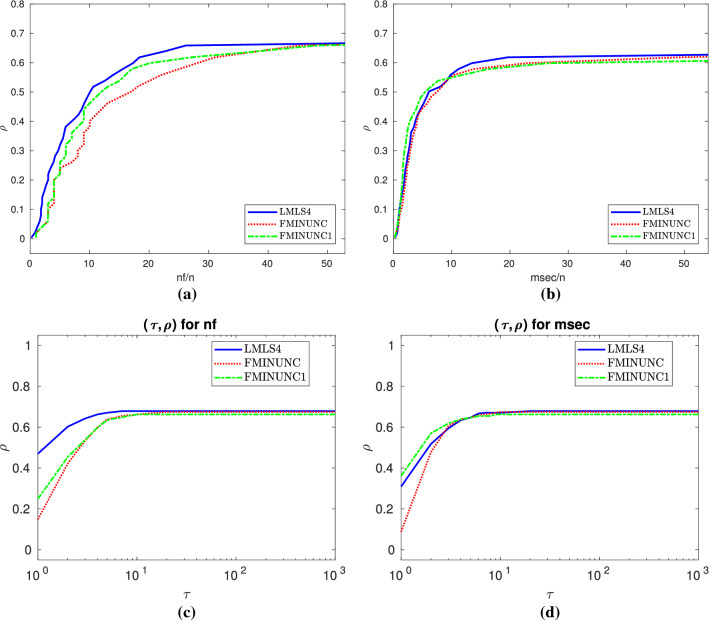
Details as in Fig. 4

**Fig. 10:**
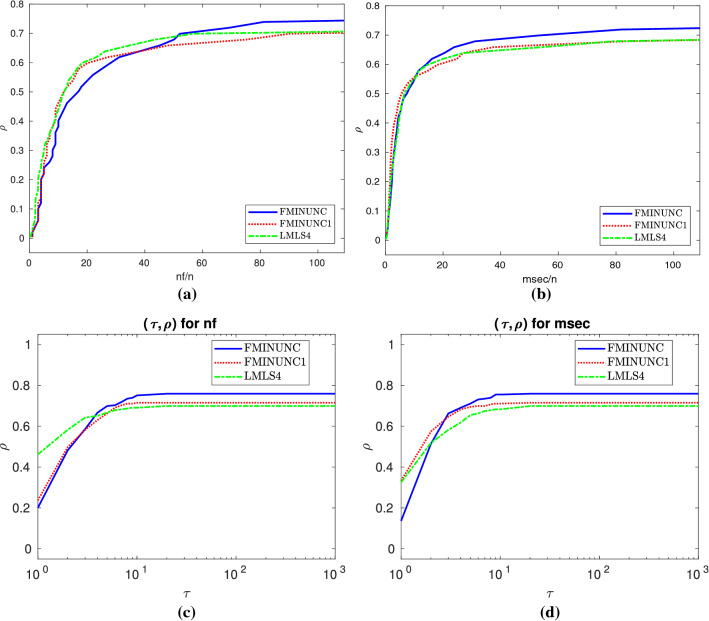
Details as in Fig. 4

This section contains Tables 6, 7, 8 and Figs. 8, 9, 10, summaries of which were discussed in Sect 3.6.

**Table 9:** Results for large-scale and small budget

Stopping test:	$q^{so}\le$ 0.001, $\mathtt{sec}\le$ 800, $\mathtt{nf}\le$ 10*n									
132 of 166 problems without bounds solved									Mean efficiency in %	
dim$\in$[1001,10000]						# of anomalies			For cost measure	
Solver		Solved	#100	!100	$T_{\text {mean}}$	#n	#t	#f	nf	msec
**LMLS4**	**lmtr4**	**101**	**47**	**44**	265873	47	17	1	**48**	**45**
**LMLS3**	**lmtr3**	97	22	19	242590	57	10	2	43	42
**LMLS2**	**lmtr2**	94	11	10	262404	60	8	4	43	41
**LMLS1**	**lmtr1**	86	22	21	302069	60	14	6	37	32
**FMINUNC1**	**func1**	81	36	35	**197750**	60	24	1	34	37

**Table 10:** Results for large-scale and budget

Stopping test:	$q^{so}\le$ 0.001, $\mathtt{sec}\le$ 800, $\mathtt{nf}\le$ 100*n									
147 of 166 problems without bounds solved									Mean efficiency in %	
dim$\in$[1001,10000]						# of anomalies			For cost measure	
Solver		Solved	#100	!100	$T_{\text {mean}}$	#n	#t	#f	nf	msec
**LMLS3**	**lmtr3**	**128**	32	29	313348	0	38	0	57	**55**
**LMLS4**	**lmtr4**	**128**	**49**	**47**	325880	0	38	0	**60**	**55**
**LMLS2**	**lmtr2**	120	17	15	306190	0	46	0	53	49
**FMINUNC1**	**func1**	104	37	36	**236336**	0	61	1	42	47
**LMLS1**	**lmtr1**	95	18	17	332592	0	71	0	39	33

**Fig. 11:**
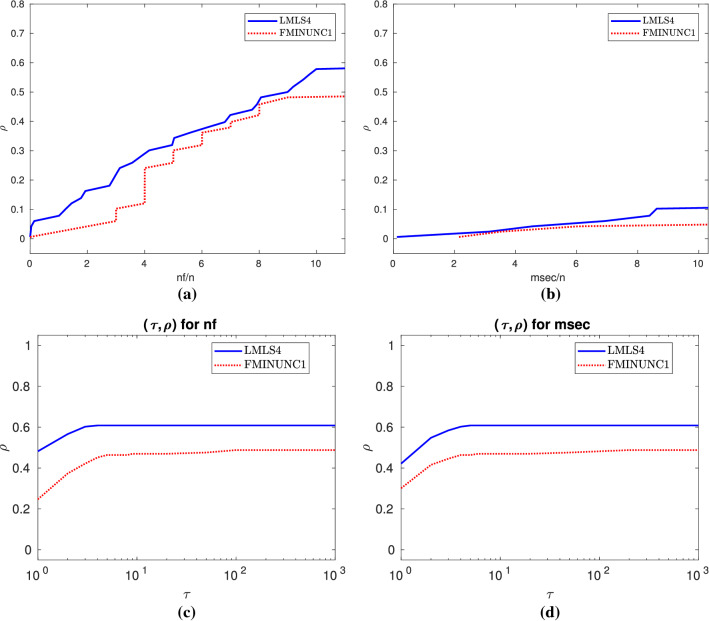
Details as in Fig. 4

### Tables and data/performance profiles for $1001 \le n \le 10000$

**Fig. 12:**
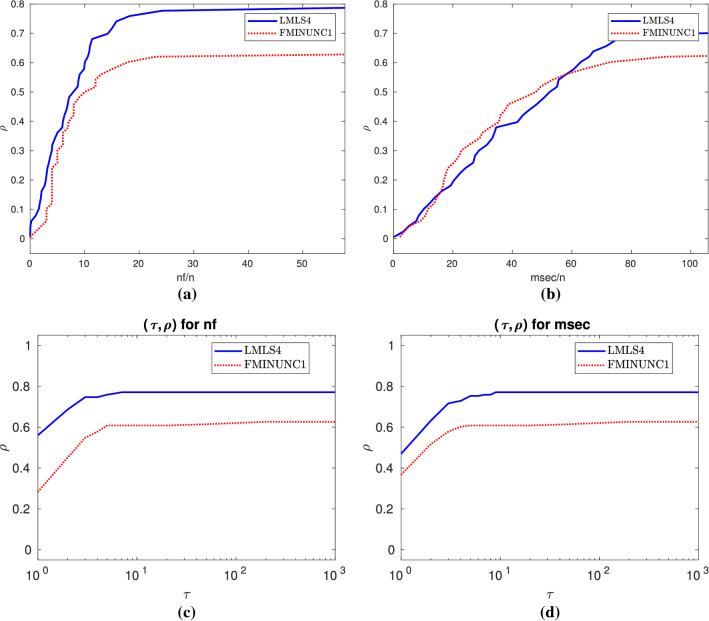
Details as in Fig. 4

This section contains Tables 9, 10 and Figs. 11, 12, summaries of which were discussed in Sect 3.7.
